# Looking into the Eyes to See the Heart of Chronic Kidney Disease Patients

**DOI:** 10.3390/life14040533

**Published:** 2024-04-22

**Authors:** Maria Kislikova, Jorge Javier Gaitán-Valdizán, José Antonio Parra Blanco, María Teresa García Unzueta, María Rodríguez Vidriales, Clara Escagedo Cagigas, Vicente Celestino Piñera Haces, María de la Oliva Valentín Muñoz, Adalberto Benito Hernández, Juan Carlos Ruiz San Millan, Emilio Rodrigo Calabia

**Affiliations:** 1 Immunopathology Group, Nephrology Department, Marqués de Valdecilla University Hospital—IDIVAL, 39008 Santander, Spain; maria.rodriguezv@scsalud.es (M.R.V.); clara.escagedo@scsalud.es (C.E.C.); vicentecelestino.pinera@scsalud.es (V.C.P.H.); mariadelaoliva.valentin@scsalud.es (M.d.l.O.V.M.); adalberto.benito@scsalud.es (A.B.H.); juancarlos.ruiz@scsalud.es (J.C.R.S.M.); emilio.rodrigo@scsalud.es (E.R.C.); 2 Ophthalmology Department, Marqués de Valdecilla University Hospital—IDIVAL, 39008 Santander, Spain; jorgejavier.gaitan@scsalud.es; 3 Radiology Department, Marqués de Valdecilla University Hospital—IDIVAL, 39008 Santander, Spain; joseantonio.parra@scsalud.es; 4 Clinical Laboratory Department, Marqués de Valdecilla University Hospital—IDIVAL, 39008 Santander, Spain; mteresa.garciau@scsalud.es

**Keywords:** CKD, choroid, retinal nerve, cardiac disease, coronary disease, troponin

## Abstract

In patients with chronic kidney disease (CKD), the main cause of morbidity and mortality is cardiovascular disease (CVD). Both coronary artery calcium scoring by computed tomography (CT) and optical coherence tomography (OCT) are used to identify patients at increased risk for ischemic heart disease, thereby indicating a higher cardiovascular risk profile. Our study aimed to investigate the utility of these techniques in the CKD population. In patients with CKD, OCT was used to measure the choroidal thickness (CHT) and the thickness of the peripapillary retinal nerve fiber layer (pRNFL). A total of 127 patients were included, including 70 men (55%) with an estimated glomerular filtration rate (eGFR) of 39 ± 30 mL/min/1.73 m^2^. Lower pRNFL thickness was found to be related to high-sensitivity troponin I (r = −0.362, *p* < 0.001) and total coronary calcification (r = −0.194, *p* = 0.032). In a multivariate analysis, pRNFL measurements remained associated with age (β = −0.189; −0.739–−0.027; *p* = 0.035) and high-sensitivity troponin I (β = −0.301; −0.259–−0.071; *p* < 0.001). Severe coronary calcification (Agatston score ≥ 400 HU) was related to a worse eGFR (*p* = 0.008), a higher grade of CKD (*p* = 0.036), and a thinner pRNFL (*p* = 0.011). The ROC curve confirmed that the pRNFL measurement could determine the patients with an Agatston score of ≥400 HU (AUC 0.638; 95% CI 0.525–0.750; *p* = 0.015). Our study concludes that measurement of pRNFL thickness using OCT is related to the markers associated with ischemic heart disease, such as coronary calcification and high-sensitivity troponin I, in the CKD population.

## 1. Introduction

Chronic kidney disease (CKD) affects between 3% and 18% of the world’s population, and its incidence has been increasing in recent years [1,2]. CKD is defined as structural or functional changes in the kidneys that persist for more than three months, according to the Kidney Disease Improving Global Outcomes (KDIGO) guidelines. After diagnosis, classifying the stage of CKD is recommended via the estimated glomerular filtration rate (eGFR) using the CKD-EPI formula and the level of albuminuria. There are five grades of CKD based on the eGFR: grade I (>90 mL/min/1.73 m^2^), grade II (60–89 mL/min/1.73 m^2^), grade III (30–60 mL/min/1.73 m^2^), grade IV (15–30 mL/min/1.73 m^2^), and grade V (<15 mL/min/1.73 m^2^). There are three grades of CKD based on albuminuria: A1 (<30 mg/g or <3 mg/mmol), A2 (30–300 mg/g or 3–30 mg/mmol), and A3 (>300 mg/g or >30 mg/mmol). The higher the level of eGFR and albuminuria, the greater the risk of complications, CKD progression, and cardiovascular disease [3,4].

CKD has a significant economic impact on health systems and contributes to a reduction in the quality of life [1,5]. Globally, approximately 3 million patients suffering from CKD grade V need renal replacement therapy. However, it is estimated that the actual number of patients requiring renal replacement therapy is around 10 million, which is alarming. Moreover, it is expected that there will be an increase of 50–100% by 2030 [6].

This population under consideration has a high risk of cardiovascular disease (CVD), which is the leading cause of death among them [7]. Approximately 50% of patients with CDK grades IV and V have CVD [8]. Chronic inflammation, endothelial dysfunction, vascular calcification, and hemodynamic changes related to cardiac disease are the key factors contributing to the pathophysiology of CVD [9,10]. Nowadays, the factors responsible for the development of CVD are classified as traditional and non-traditional atherosclerosis risk factors. Traditional risk factors include age, sex, dyslipidemia, arterial hypertension (HT), diabetes mellitus (DM), and smoking habits [11,12]. Non-traditional risk factors include bone metabolism, chronic inflammation, and anemic status [11,12].

Other important risk factors for the progression of CKD are HT and DM, with the estimated prevalence of HT being 32% in women and 34% in men, and 10% prevalence for DM [13,14]. Both of these systemic diseases have a strong association with CVD, and their interrelationship contributes to cardiovascular morbidity and premature mortality in CKD patients [1].

Microvascular changes are very important in the process of CKD development and progression. Alterations in microvascular structure and function contribute to the development and progression of hypertension, diabetes, CKD, and CVD [15,16]. At present, these microvascular changes can be only accessed via kidney biopsy, but that is an aggressive procedure, and, as Farrah et al. highlighted, novel biomarker development is an unmet necessity for tracking kidney injury [7]. Ideal biomarkers would be noninvasive, cheap, and not time-consuming. Specifically, noninvasive methods in early-stage CKD could offer significant clinical utility for identifying and tracking. It might be easier, based on the follow-up of these methods, to offer interventions to reduce renal decline before irreversible end-organ damage is done [17].

In recent years, studies showing similarities between the kidney and the eyes have been published [18]. The eye is considered a window to the kidney because of the transparent ocular media that allow a direct visualization of the microvasculature [19]. It is possible to see and measure the whole retina and its multiple layers using noninvasive optical coherence tomography (OCT). The peripapillary retinal nerve fiber layer (pRNFL) mirrors retinal ganglion cell axon damage, which can be detected on examination of the fundus via OCT and results in optic disk pallor and/or excavation. RNFL thinning reflects the axonal loss within the optic nerve resulting from injuries within the retina, the optic nerve, the optic chiasm, or the optic tracts caused by different local or systemic diseases [20]. The choroid is a network of blood vessels, collagen fibers, fibroblasts, and melanocytes, and changes in choroidal thickness are believed to be an indicator of the impact of systemic diseases on the vascular aspects of the eye. Choroidal thickness has been found altered in different subclinical diseases such as arterial hypertension [21], chronic kidney disease, and diabetes; and in clinical diseases such as age-related macular disease, pachychoroid spectrum of diseases, and choroiditis (inflammatory or infectious) [22].

In a study of patients with CKD but without DM, the pRNFL and macular thickness were found significantly lowered compared with the healthy population [23]. More recently, it was reported that patients with CKD experienced faster pRNFL loss compared with healthy controls, and the loss of the pRNFL was associated with the severity of CKD and HT [24]. Balmforth et al. found that chorioretinal thinning in CKD is associated with lower eGFR and greater proteinuria [25]. The retinal and choroidal thickness were reduced depending on the stage of CKD: greater reduction in CKD stages 3–5 compared with CKD stages 1–2. In the same study, a thinner retina and choroid were associated with an elevated serum C-reactive protein concentration and higher proteinuria [26]. The thickness of the retinal layers has been associated with several chronic conditions, including DM and HT, which are indeed considered risk factors for CKD [27,28].

The leading cause of death in the CKD population is a consequence of CVD, including coronary artery disease (CAD) [29]. Noninvasive modalities play a crucial role in evaluating CAD. An important noninvasive method to study macrovascular changes is the quantification of coronary artery calcification (CAC) via a computed tomography (CT) scan, and this is a good marker of ischemic heart disease in the general and CKD populations [30]. Functional assessments via echocardiography and cardiac magnetic resonance assess myocardial perfusion and wall motion abnormalities, which can aid in the diagnosis of ischemia and its impact on heart function. Although not directly indicative of calcification or ischemia, troponin blood tests can support a diagnosis when elevated levels suggest heart muscle damage. Cardiac troponin is an essential protein found in the myocytes, and it is known that changes in high-sensitivity troponin I provide prognostic value for both sudden cardiac death and all-cause mortality [31]. Another important blood test is NT-ProBNP, which indicates heart failure. Lastly, increased suppression of the level of tumorigenicity-2 (ST2) was found to be associated with increased risk of cardiovascular events and/or death in kidney transplant candidates [32].

Patients affected by CAD were discovered to have significantly thinner choroids than healthy controls [33]. In another study, Kocamaz et al. confirmed that patients with CAD have a decreased choroidal thickness compared with patients at risk of CAD in the general population [34]. There has only been one study conducted in the CKD population that demonstrated no association between CKD and CAD, and none was found using retinal vessel caliber in an adjusted model [35]. Until now, no study has been conducted to ascertain whether any relationship exists between the microvascular damage seen using retinal vessel measurements and CAC in the CKD population.

Our aim was to analyze the relationship of CHT and pRNFL with renal function and silent coronary disease in a CKD population without previously known CVD.

## 2. Materials and Methods

Adult CKD patients (older than 18 years) being monitored in nephrology clinics were included in the study between 1 September 2020 and 31 January 2021 if they were willing to participate and sign the informed consent. The exclusion criteria were previous cardiovascular pathology, confirmed or symptomatic cardiac disease, and atrial fibrillation. In total, 127 patients gave their consent to participate in the study. The study was approved by the Cantabria Clinical Research Ethics Committee (2020:326).

Together with the collection of anthropometric and clinical data, blood samples were taken in a standardized manner, from which creatinine (eGFR using the CKD-EPI equation) was immediately analyzed. In addition, a frozen serum sample was kept at −80 °C for specific determinations. Troponin I (high-sensitivity troponin I) and Pro-BNP (NT-proBNP) were measured using an automated immunoassay on an Atellica^®^ IM (Siemens^®^ Healthineers, Tarrytown, NY, USA).

Coronary artery calcification obtained via computed tomography using 64/128 Optima (64) and Revolution EVO (128) detectors (GE Healthcare, Chicago, IL, USA) was measured using the prospective acquisition technique with heart rate monitoring. The amount of calcium in the coronary arteries was measured using SmartScore^®^ software (General Electric Healthcare, USA), and the amount of calcium was expressed according to the system proposed by Agatston. Agatston’s classification is defined as 0–100, 100–200, and over 400 HU, etc. To carry out the study, the total amount of coronary calcium (total-CAC) in all coronary arteries was evaluated, and the amounts for the left anterior descending artery (LAD-CAC) and the circumflex coronary artery (Cx-CAC) were separately evaluated.

**Figure 1:**
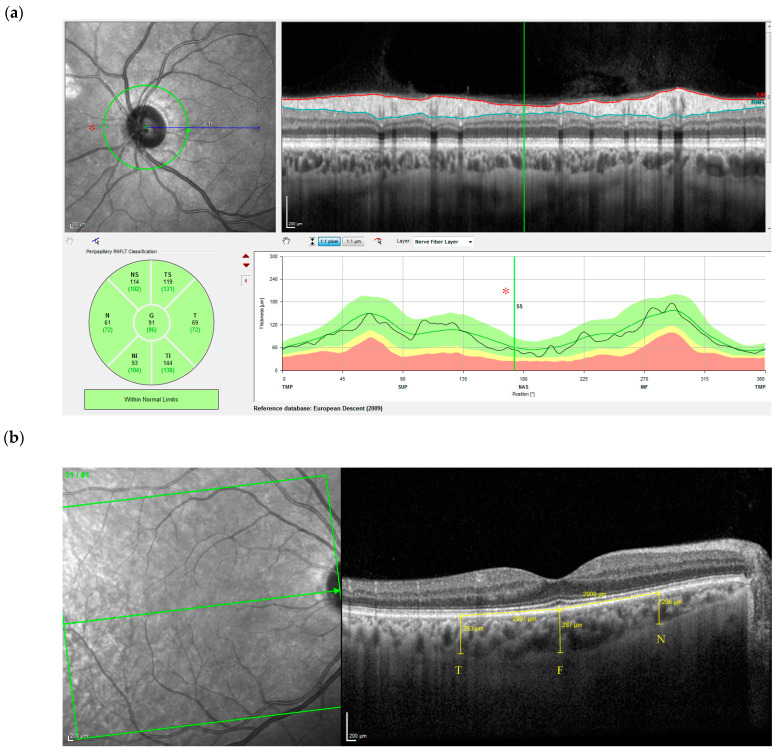
Chorioretinal structures. (**a**) Left up image: A circular green grid is centered over the optic nerve and consists of one concentric ring 3.5 mm in diameter around the optic nerve head. The blue line represents the projection of the center of this ring to the fovea. The ring is divided into six quadrants: temporal (T), temporal-superior (TS), temporal-inferior (TI), nasal (N), nasal-superior (NS), nasal-inferior (NI), and global (G). Scale bars: 200 μm. Right up image: Cross-sectional scan presented in a horizontal diagram in which the extreme ends of the diagram represent the temporal retinal nerve fiber, blue line is the retinal nerve fiber layer thickness, red line is the internal limiting membrane, green line corresponds to the position of the red asterisk shown in left up and right down image. Left down image: The study map divides the macula into six subfields and the global in the middle. Right down image: The quantification of the thickness of the retinal ganglion cell axons where white would be above normal, green is within normal range, yellow is borderline, and red is clearly reduced compared with normal. (**b**) Left image: OCT of fundus image, Right image is corresponding OCT Image below the green arrow inside the green box. Choroidal thickness was measured at three locations on the macula (yellow lines): N = 2000 μm nasal to the fovea, F = subfoveal, and T = 2000 μm temporal to the fovea. Scale bars: 200 μm.

The OCT test was performed by the Ophthalmology Department of University Hospital Marqués de Valdecilla, with images obtained using the OCT Spectralis HRA + machine (Heidelberg Engineering, Heidelberg, Germany). The scanning wavelength used was 870 nm. During the examination, the patients remained seated, and the use of any type of medication in the form of eye drops was not essential for the examination. The image acquired was a depth-enhanced image averaging approximately 100 slices using the automatic mode. The CHT was measured at various locations on the retina of the right eye: in the fovea, 2000 μm nasal to the fovea, and 2000 μm temporal to the fovea. The pRNFL parameters included in the analysis were peripapillary thickness measurements of mean global thickness and in each of the six sectoral measurements (temporal-superior, temporal, temporal-inferior, nasal-inferior, nasal, and nasal-superior) in the right eye (Figure 1).

### Statistical Analysis

Continuous variables are expressed as the mean and standard deviation. Categorical variables are described as relative frequencies. The relationships between pRNFL or CHT and the continuous variables were analyzed using Pearson correlation for the continuous variables and the chi-square test for the categorical variables. Subsequently, to evaluate the relationship among the continuous variables, a univariate linear regression analysis was performed. To explore which variables were independently related, multivariate linear regression analysis was carried out. Univariate and multivariate logistic regression was conducted to study the variables related to an Agatston value ≥ 400 HU. The ability of the pRNFL to discriminate which patients had coronary calcification with an Agatston score of ≥400 HU was explored using the ROC curve. All statistical analyses were performed using the SPSS version 15.0 program (SPSS, Inc., Chicago, IL, USA), and *p* < 0.05 was considered statistically significant.

## 3. Results

A total of 127 patients were included, including 70 men (55%) with a eGFR of 39 ± 30 mL/min/1.73 m^2^. The etiology of CKD in our population was as follows: glomerular (23%), diabetic (17%), vascular (15%), hereditary (9%), interstitial (9%), systemic disease (6%), and unknown cause of CKD (21%)**.** Of our patients, 110 (87%) had HT and most of them were treated with three drugs (26%), and the most frequently used drugs were renin–angiotensin–aldosterone system inhibitors (51%). Other patient characteristics are shown in Table 1.

### 3.1. pRNFL

The mean value of the pRNFL was 95 ± 14 μm. There were no differences in pRNFL thickness between men and women: 93 ± 14 μm vs. 97 ± 15 μm, *p* = 0.102. There were also no differences between diabetics and non-diabetics: 93 ± 15 μm vs. 96 ± 14 μm, *p* = 0.251. No difference was detected between smokers and non-smokers: 96 ± 11 μm vs. 92 ± 20 μm, *p* = 0.258.

The degree of pRNFL thinning was related to increasing age and the use of more antihypertensive drugs for hypertension control. However, the association between HT and pRNFL was not found to be significant: *p* = 0.839. The eGFR was measured using the CKD-EPI equation such that those patients with a lower eGFR had a thinner pRNFL. All the evaluated parameters of coronary calcification had a confirmed inverse correlation with the thickness of pRNFL, so those patients with more calcification had a thinner pRNFL. The higher the total CAC, the more pRNFL thinning was detected, and the same occurred with LAD-CAC and Cx-CAC, respectively. We found a correlation between a higher level of high-sensitivity troponin I concentration and a thinner pRNFL, but these findings were not confirmed with NT-ProBNP. Interestingly, the degree of pRNFL thinning was related to lower triglyceridemic levels. Details of all the studied parameters with Pearson correlation can be found in Table 2.

Using univariate linear regression analysis, a lower eGFR was found to be significantly associated with a thinner pRNFL (β = 0.171; −0.003–0.162; *p* = 0.058). The association did not remain significant in the multivariate analysis (adjustments were made for parameters with *p* < 0.05 in the univariate model: eGFR, age, HT). The hypertensive patients had a thinner pRNFL in the univariate linear regression analysis (β =−0.231; −16.961–2.437; *p* = 0.009), but the association did not remain significant in the multivariate model. An association was also seen between elderly patients and a thinner pRNFL (β = −0.212; −0.786–−0.074; *p* = 0.018), and between lower triglyceridemic levels and a thinner pRNFL (β = 0.189; 0.002–0.071; *p* = 0.036) in the unadjusted model. However, only the age (β = −0.189; −0.739–−0.027; *p* = 0.035) and the high-sensitivity troponin I (β = −0.301; −0.259–−0.071; *p* < 0.001) maintained significance after adjustment (Table 3).

Almost all the parameters for silent cardiac disease were associated with a lower pRNFL. A higher level of high-sensitivity troponin I was associated with a thinner pRNFL (β = −0.366; −0.286–−0.107; *p* < 0.001) in the unadjusted analysis. In the multivariate model, the association remained significant (β = −0.301; −0.259–−0.071; *p* < 0.001), and adjustments were made for the parameters with *p* < 0.05 in the univariate model: age and CAC. This association was also seen with CAC. The higher the total-CAC, the greater the pRNFL thinning detected (β = −0.194; −0.004–0.000; *p* = 0.032), and the same tendency occurred with LAD-CAC (β =−0.245; −0.015–−0.003; *p* = 0.006) and Cx-CAC (β = −0.216; −0.026–0.003; *p* = 0.016) in the unadjusted analysis. The total-CAC association was no longer significant following adjustment, and the separated analysis of arteries association was not maintained either. There was no association between the pRNFL and NT-ProBNP in the unadjusted and adjusted models (Table 3).

### 3.2. CHT

The mean value of CHT was 247 ± 81 μm. There were no differences in CHT thickness observed between men and women: 241 ± 79 μm vs. 254 ± 85 μm, *p* = 0.372. No differences were detected between diabetics and non-diabetics: 243 ± 82 μm vs. 249 ± 81 μm, *p* = 0.721, and none between smokers and non-smokers: 239 ± 79 μm vs. 250 ± 83 μm, *p* = 0.446. The relationship between CHT and the continuous variables is shown in Table 2. The degree of CHT thinning was related to increasing age and a lower eGFR, as measured by CKD-EPI equation, such that those patients with a lower eGFR had a thinner CHT. Higher C-reactive protein levels (CRP) were associated with a greater degree of CHT thinning (r = −0.215, *p* = 0.016). There was no association seen between HT and CHT (*p* = 0.438), nor between a number of antihypertensive drugs and CHT (*p* = 0.124). Interestingly, a decreasing thickness of CHT was related to lower PTH levels (r = 0.214, *p* = 0.016). All of the evaluated parameters of silent cardiac disease were related to CHT. Details of all the parameters studied with Pearson correlation can be found in Table 2.

CHT changes were associated with CKD. A thinner CHT was associated with a better eGFR, as measured by the CKD-EPI equation (β = −0.190; −0.977–−0.040; *p* = 0.034) in the unadjusted analysis. It did not remain significantly associated following an adjustment for the parameters with *p* < 0.05 in the univariate model: eGFR, age, CRP, and PTH. An association between elderly patients (β = −0.288; −5.340–−1.363; *p* = 0.001) and higher CRP levels (β = −0.204; −35.699–−2.760; *p* = 0.022) with reduced CHT was seen in the unadjusted model. Both of these were maintained as significant in the adjusted analysis [age (β = −0.221; −4.556–−0.574; *p* = 0.012) and CRP levels (β = −0.206; −35.267–−3.608; *p* = 0.017)]. Any of the evaluated parameters of silent cardiac disease were found to be significant in the linear regression using the unadjusted or adjusted model for CHT (Table 3).

### 3.3. Severe Calcification and Ophthalmologic Parameters

Table 4 shows an analysis of the variables related to the severe (Agatston value ≥ 400 HU) global coronary calcification found in 39 patients (31%). Severe global coronary calcification is related to a lower eGFR and to end-stage CKD. The patients with an Agatston score of ≥400 HU were more likely to have DM (*p* < 0.001) and hyperphosphatemia. Both of the studied biochemical cardiac parameters (high-sensitivity troponin I and NT-ProBNP) were found to be higher in the patients with severe global coronary calcification.

**Figure 2:**
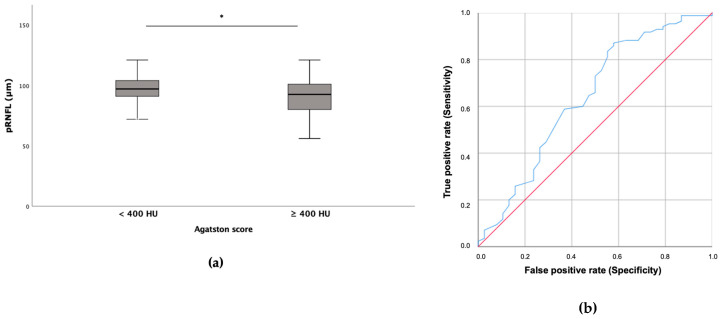
The relationship between pRNFL and severe coronary disease. (**a**) Patients with severe global coronary calcification (≥400 HU) had a thinner pRNFL (89 ± 17 μm) than those with non-severe global coronary calcification (<400 HU), who had a pRNFL of 97 ± 13 μm (* *p* = 0.011). (**b**) ROC curve showing that pRNFL thickness could indicate those patients with an Agatston score of ≥400 HU (AUC 0.638; 95% CI 0.525–0.750; *p* = 0.015). Blue line—ROC curve, red line—baseline.

The patients with severe global coronary calcification had a thinner pRNFL (Figure 2a). To confirm these results, a ROC curve was performed, showing that the pRNFL measurement could determine the patients with an Agatston score of ≥400 HU (AUC 0.638; 95% CI 0.525–0.750; *p* = 0.015) (Figure 2b). By contrast, no relationship was found with CHT (Table 4).

The odds ratios (OR) and 95% confidence intervals (CI) of pRNFL thickness or CHT for those with a high Agatston score were estimated. Since we had 39 patients in the group with severe coronary calcification, we conducted three models of multivariate analysis adjustment with the different parameters that were seen as significant in the univariate analysis. The first model was adjusted for eGFR, HT, and hyperphosphatemia, and the patients with severe global coronary calcification were found to have a thinner pRNFL (OR 0.967; 95% CI 0.937–0.997; *p* = 0.032). The second model was adjusted for high-sensitivity troponin I, NT-ProBNP, and LDL-cholesterol, and the patients with severe global coronary calcification also had a thinner pRNFL (OR 0.967; 95% CI 0.936–1.000; *p* = 0.047). The third model was adjusted for DM, NT-ProBNP, and HT, and the patients with severe global coronary calcification had a thinner pRNFL (OR 0.964; 95% CI 0.933–0.996; *p* = 0.029) (Table 5).

## 4. Discussion

Changes in microvascular structure and function are usually caused by hypertension, diabetes, and CKD, with a later association with cardiovascular disease. Similarities between vessels of the eye and the kidney suggest that noninvasive imaging of the retinal vessels can detect these microvascular alterations and could be added to the current renal and cardiovascular risk stratification tools [19]. Nowadays, it is well known that some optic neuropathies, such as ischemic optic neuropathy, uremic optic neuropathy, glaucoma, and macular thinning, are linked to CKD [36,37,38]. Moreover, it seems that pRNFL thickness and CHT can be considered new noninvasive biomarkers for CVD risk stratification. To the best of our knowledge, this is the first study demonstrating an association between the pRNFL and silent cardiac disease in the CKD population.

Possible reasons for pRNFL thinning in these patients include subclinical uremic optic neuropathy and chronic vascular insufficiency related to CKD-induced complications such as anemia, hypertension, or atherosclerosis [39,40]. Because of the above-mentioned microvascular injuries and choroidal thinning in CKD patients, the supply of blood can be compromised, and this can cause retinal atrophy [25,41,42]. This fact is supported by observations of decreased retinal vessel density and parafoveal retinal thickness in patients with CKD [43,44]. We observed a correlation between a thinner pRNFL and a higher stage of CKD, and, as expected, those with end-stage kidney failure had the thinnest pRNFL. This finding is in accordance with the previous literature. Paterson et al. reported the same association with advanced CKD in patients with cardiovascular risk factors or cardiovascular disease independently of important confounding factors [45]. The same association was also confirmed in the diabetic population [46]. Mule et al. reported reduced CHT in the population with CKD, and there was a similar tendency shown in the diabetic population [46,47]. In our study, an increased CHT was detected in the low eGFR patients. Balmoforth et al. found increasing age was associated with reduced CHT as well as higher CRP concentrations, findings that we confirmed [25].

On one hand, our results confirm previously published data related to CKD and pRNFL measurements and their relationship with age and CKD stage. On the other hand, the main finding of our study was the relationship between pRNFL and silent cardiac disease in CKD patients. We reported, for the first time, the correlation between silent cardiac disease in the CKD population and pRNFL. In patients with CKD, a thinning pRNFL is associated with total coronary calcification. In the separated analysis for LAD and CCA, the same statistically significant inverse correlations between a thinner pRNFL and higher levels of coronary calcification were seen. Similarly, measurement of high-sensitivity troponin I concentration as a blood parameter of cardiac disease was statistically significantly higher with thinning pRNFL. It is well known that a high concentration of troponin is related to ischemic heart disease, unlike high concentrations of NT-ProBNP being related to cardiac dysfunction.

When we analyzed our data by dividing our population into non-severe coronary calcification and severe coronary calcification (Agatston score ≥ 400 HU), the population with severe coronary calcification had a significantly thinner pRNFL. Moreover, AUC-ROC analysis suggested that the pRNFL could indicate which patients showed silent coronary disease.

Our study is a prospective study and a perfect follow-up, without the loss of any patients. We consider these to be the strengths of our study. In future, these noninvasive measurements using widely available and noninvasive technology might help us to achieve better cardiovascular risk stratification of our CKD population.

The study patient data came from only one center, and the study reflects the population of Spain (most of the patients were Caucasian), which makes the sample less comparable with the global population. It is quite a small sample if we consider the prevalence of CKD in the general population. We have 37% of patients with DM who could have changes seen using OCT based on diabetic retinopathy, not only related to the aim of our study. On the other hand, we do not have available data on the diagnosis of glaucoma in our population. It is well known that 50% of the population with glaucoma are undiagnosed and that glaucoma produces increased CHT and RNLF thickening. During our study, data regarding medications were not collected; however, in future studies, it would be important to analyze this because of its relationship with calcification. Longitudinal studies may provide greater information about the noninvasive imaging of the retinal vessel changes that occur in CKD.

## 5. Conclusions

The measurement of pRNFL thickness using OCT may enable noninvasive cardiovascular risk stratification for patients with CKD. A lower pRNFL thickness is evident in 64% of patients with severe coronary calcification. In our study, the measurement of pRNFL thickness was related to markers of ischemic heart disease, such as severe coronary calcification and high-sensitivity troponin I, in the CKD population. In different models, we were able to show that patients with severe global coronary calcification had a thinner pRNFL independently of some of the atherosclerosis risk factors (HT, LDL-cholesterol, DM, hyperphosphatemia) and blood tests related to ischemic and functional cardiac damage (high-sensitivity troponin I, NT-ProBNP). OCT can be easily performed by any ophthalmology department; it is increasingly used in clinical practice, regular software is used for the automatic measurement of pRNFL, and it can be carried out easily, quickly, and without any patient preparation. We consider this measurement low in cost because no additional expenditure is needed for typical healthcare units with an ophthalmology department. Therefore, we conclude that studying patients with CKD using OCT may allow for a noninvasive estimation of cardiovascular risk.

## Figures and Tables

**Table 1:** Participant characteristics.

Studied Variables	Value
All participants (*n*)	127
Male (*n*, %)	70 (55%)
Age (years, mean ± SD)	61 ± 7
DM (*n*, %)	47 (37%)
HT (*n*, %)	110 (87%)
Non-smokers (*n*, %)	40 (31%)
eGFR (mL/min/1.73 m^2^, mean ± SD)	39 ± 30
BMI (kg/m^2^, mean ± SD)	28 ± 5
C-reactive protein (mg/dL, mean ± SD)	0.6 ± 0.8
High-sensitivity troponin I (ng/L, mean ± SD)	16 ± 26
NT-ProBNP (pg/mL, mean ± SD)	2310 ± 6460
Triglycerides (mg/dL, mean ± SD)	143 ± 72
LDL cholesterol (mg/dL, mean ± SD)	94 ± 39
HDL cholesterol (mg/dL, mean ± SD)	48 ± 16
Calcium (mg/dL, mean ± SD)	9 ± 0.7
Phosphate (mg/dL, mean ± SD)	4 ± 1.3
PTH (pg/mL, mean ± SD)	212 ± 245
Vitamin D (ng/mL, mean ± SD)	20 ± 10
CAC—total (HU, mean ± SD)	544 ± 1251
CAC—LAD (HU, mean ± SD)	223 ± 383
CAC—CCA (HU, mean ± SD)	77 ± 213
CHT (μm, mean ± SD)	247 ± 81
pRNFL (μm, mean ± SD)	95 ± 14

DM = diabetes mellitus, HT = arterial hypertension, eGFR = estimated glomerular filtration rate by CKD-EPI form, BMI = body mass index, PTH = parathyroid hormone, LDL = low-density lipoprotein, HDL = high-density lipoprotein, NT-ProBNP = plasma N-terminal probrain natriuretic peptide, CAC = coronary artery calcification, CAC—LAD = coronary artery calcification of left anterior descending artery, CAC—CCA = coronary artery calcification of circumflex coronary artery, CHT = choroidal thickness, pRNFL = thickness of the peripapillary retinal nerve fiber layer.

**Table 2:** Pearson correlation between pRNFL or CHT and continuous variables.

	pRNFL		CHT	
	r	*p*	r	*p*
eGFR (mL/min/1.73 m^2^)	0.177	0.049 *	−0.181	0.043 *
Age (years)	−0.216	0.016 *	−0.291	0.001 *
CRP (mg/dL)	−0.066	0.459	−0.215	0.016 *
BMI (kg/m^2^)	0.051	0.571	0.146	0.103
Number of antihypertensive drugs	−0.210	0.020 *	0.138	0.124
Triglycerides (mg/dL)	0.187	0.037 *	0.165	0.064
LDL cholesterol (mg/dL)	0.070	0.437	0.006	0.949
HDL cholesterol (mg/dL)	0.031	0.732	−0.104	0.248
PTH (pg/mL)	−0.058	0.526	0.214	0.016 *
Vitamin D (ng/mL)	0.017	0.855	−0.053	0.556
Phosphate (mg/dL)	−0.101	0.264	0.086	0.338
Calcium (mg/dL)	0.016	0.863	−0.041	0.358
High-sensitivity troponin I (ng/L)	−0.362	<0.001 *	−0.161	0.072
NT-ProBNP (pg/mL)	−0.063	0.449	−0.093	0.302
CAC—total (HU)	−0.194	0.032 *	−0.184	0.648
CAC—LAD (HU)	−0.245	0.006 *	−0.004	0.964
CAC—CCA (HU)	−0.216	0.016 *	0.020	0.827

eGFR = estimated glomerular filtration rate by CKD-EPI form, CRP = C-reactive protein levels, BMI = body mass index, LDL = low-density lipoprotein, HDL = high-density lipoprotein, PTH = parathyroid hormone, NT-ProBNP = plasma N-terminal probrain natriuretic peptide, CAC = coronary artery calcification, CAC—LAD = coronary artery calcification of left anterior descending artery, CAC—CCA = coronary artery calcification of circumflex coronary artery, CHT = choroidal thickness, pRNFL = thickness of the peripapillary retinal nerve fiber layer. * significant *p*-value.

**Table 3:** Univariate and multivariate linear regression. (**a**) Analysis conducted on pRNFL. (**b**) Analysis conducted on CHT.

(a)	pRNFL					
	Univariate			Multivariate		
	β	95% CI	*p*	β	95% CI	*p*
eGFR (mL/min/1.73 m^2^)	0.171	−0.003–0.162	0.058 *	0.128	−0.026–0.144	0.170
Age (years)	−0.212	−0.786–−0.074	0.018 *	−0.189	−0.739–−0.027	0.035 *
C-reactive protein (mg/dL)	0.049	−2.120–3.727	0.587	-	-	-
BMI (kg/m^2^)	0.055	−0.331–0.623	0.546	-	-	-
HT	−0.231	−16.961–2.437	0.009 *	−0.151	−13.771–1.406	0.109
Triglycerides (mg/dL)	0.189	0.002–0.071	0.036 *	0.148	−0.004–0.063	0.083
LDL cholesterol (mg/dL)	0.067	−0.041–0.090	0.459	-	-	-
HDL cholesterol (mg/dL)	0.030	−0.136–0.190	0.743	-	-	-
PTH (pg/mL)	−0.032	−0.012–0.009	0.725	-	-	-
Vitamin D (ng/mL)	0.013	−0.228–0.265	0.884	-	-	-
Calcium (mg/dL)	0.004	−3.529–3.710	0.961	-	-	-
Phosphate (mg/dL)	−0.099	−2.965–0.851	0.275	-	-	-
High-sensitivity troponin I (ng/L)	−0.366	−0.286–−0.107	<0.001 *	−0.301	−0.259–−0.071	<0.001 *
NT-ProBNP (pg/mL)	−0.063	−0.001–0.000	0.486	-	-	-
Total-CAC (HU)	−0.194	−0.004–0.000	0.032 *	−0.119	−0.003–0.001	0.166
LAD-CAC (HU)	−0.245	−0.015–−0.003	0.006 *	−0.152-	−0.012–0.001	0.085
Cx-CAC (HU)	−0.216	−0.026–0.003	0.016 *	−0.142	−0.021–0.002	0.102
**(b)**	**CHT**					
	**Univariate**			**Multivariate**		
	**β**	**95% CI**	***p***	**β**	**95% CI**	***p***
eGFR (mL/min/1.73 m^2^)	−0.190	−0.977–−0.040	0.034 *	−0.120	−0.831–0.191	0.217
Age (years)	−0.288	−5.340–−1.363	0.001 *	−0.221	−4.556–−0.574	0.012 *
C-reactive protein (mg/dL)	−0.204	−35.699–−2.760	0.022 *	−0.206	−35.267–−3.608	0.017 *
BMI (kg/m^2^)	0.150	−0.398–4.960	0.094	-	-	-
HT	0.065	−27.050–57.572	0.477	-	-	-
Triglycerides (mg/dL)	0.167	−0.010–0.388	0.062	-	-	-
LDL cholesterol (mg/dL)	0.002	−0.369–0.379	0.980	-	-	-
HDL cholesterol (mg/dL)	−0.106	−1.397–0.353	0.240	-	-	-
PTH (pg/mL)	0.260	0.029–0.144	0.003 *	0.179	−0.006–0.125	0.074
Vitamin D (ng/mL)	−0.057	−1.846–0.953	0.529	-	-	-
Calcium (mg/dL)	−0.096	−31.881–9.464	0.285	-	-	-
Phosphate (mg/dL)	0.089	−5.460–16.453	0.323	-	-	-
High-sensitivity troponin I (ng/L)	−0.165	−1.052–0.033	0.065	-	-	-
NT-ProBNP (pg/mL)	−0.094	−0.003–0.001	0.298	-	-	-
Total-CAC (HU)	−0.041	−0.014–0.009	0.648	-	-	-
LAD-CAC (HU)	−0.004	−0.039–0.037	0.964	-	-	-
Cx–CAC (HU)	0.020	−0.061–0.076	0.827	-	-	-

eGFR = estimated glomerular filtration rate by CKD-EPI form, BMI = body mass index, HT = arterial hypertension, NT-ProBNP = plasma N-terminal probrain natriuretic peptide, LDL = low-density lipoprotein, HDL = high-density lipoprotein, PTH = parathyroid hormone, CAC = coronary artery calcification, LAD-CAC = coronary artery calcification of left anterior descending artery, Cx-CAC = coronary artery calcification of circumflex coronary artery, CHT = choroidal thickness, pRNFL = thickness of the peripapillary retinal nerve fiber layer. * significant *p*-value.

**Table 4:** Analysis between severe (Agatston score ≥ 400 HU) and non-severe global coronary calcification (Agatston score < 400 HU).

	Agatston <400 HU (*n* = 88)	Agatston ≥400 HU (*n* = 39)	*p*
eGFR (mL/min/1.73 m^2^)	42.8 ± 31.1	28.2 ± 26.4	0.008 *
CKD stage V	25.0%	43.6%	0.036 *
Age (years)	60 ± 7	62 ± 7	0.071
Sex (male)	50%	67%	0.082
BMI (kg/m^2^)	28.0 ± 5.4	29.0 ± 5.3	0.351
DM	23.9%	66.7%	<0.001 *
HT	83%	95%	0.069
Smoking (current-ex)	27.3%	41.0%	0.124
C-reactive protein (mg/dL)	0.5 ± 0.4	0.6 ± 0.9	0.258
Triglycerides (mg/dL)	146 ± 73	141 ± 71	0.701
LDL cholesterol (mg/dL)	99.3 ± 41.4	81.5 ± 28.7	0.006 *
HDL cholesterol (mg/dL)	50.0 ± 17.4	44.6 ± 13.3	0.060
PTH (pg/mL)	187.1 ± 187	258.6 ± 332.5	0.125
Vitamin D (ng/mL)	21.8 ± 9.4	18.9 ± 12.1	0.144
Calcium (mg/dL)	9.1 ± 0.7	9.1 ± 0.7	0.755
Phosphate (mg/dL)	3.8 ± 1.0	4.7 ± 1.7	0.004 *
High-sensitivity troponin I (ng/L)	12.0 ± 19.3	25.0 ± 36.1	0.040 *
NT-ProBNP (pg/mL)	1049.7 ± 2110.6	5157.2 ± 10,589.7	0.021 *
pRNFL (μm)	97 ±13	89 ± 17	0.011 *
CHT (μm)	254 ± 78	234 ± 88	0.208

eGFR = estimated glomerular filtration rate by CKD-EPI form, CKD = chronic kidney disease, BMI = body mass index, DM = diabetes mellitus, HT = arterial hypertension, LDL = low-density lipoprotein, HDL = high-density lipoprotein, PTH = parathyroid hormone, NT-ProBNP = plasma N-terminal probrain natriuretic peptide, pRNFL = thickness of the peripapillary retinal nerve fiber layer, CHT = choroidal thickness. * significant *p*-value.

**Table 5:** Analysis of multivariate logistic regression between severe (Agatston score ≥ 400 HU) and non-severe global coronary calcification (Agatston score < 400 HU), and three different models adjusted for the significant parameters from the univariate analysis.

	Multivariate Logistic Regression with Agatston Score		
	OR	95% CI	*p*
Model 1			
Adjusted for:			
• eGFR	0.998	0.980–1.016	0.820
• HT	2.063	0.406–10.482	0.382
• hyperphosphatemia	1.615	1.083–2.408	0.019 *
• pRNFL	0.967	0.937–0.997	0.032 *
Model 2			
Adjusted for:			
• high-sensitivity troponin I	1.008	0.992–1.025	0.333
• NT-ProBNP	1.000	1.000–1.000	0.052
• LDL-cholesterol	0.987	0.987–1.000	0.058
• pRNFL	0.967	0.936–1.000	0.047 *
Model 3			
Adjusted for:			
• DM	5.462	2.196–13.586	<0.001 *
• NT-ProBNP	1.000	1.000–1.000	0.041 *
• HT	1.314	0.253–6.816	0.745
• pRNFL	0.964	0.933–0.996	0.029 *

OR = odds ratio, pRNFL = thickness of the peripapillary retinal nerve fiber layer, eGFR = estimated glomerular filtration rate by CKD-EPI form, HT = arterial hypertension, NT-ProBNP = plasma N-terminal probrain natriuretic peptide, DM = diabetes mellitus, LDL = low-density lipoprotein. * significant *p*-value.

## Data Availability

The raw data supporting the conclusions of this article will be made available by the authors on request.
